# HBV DNA levels impact the prognosis of hepatocellular carcinoma patients with microvascular invasion

**DOI:** 10.1097/MD.0000000000016308

**Published:** 2019-07-05

**Authors:** Lian Li, Bo Li, Ming Zhang

**Affiliations:** a Department of Liver Surgery, West China Hospital, Sichuan University, Chengdu; b Department of General Surgery, Mianzhu Hospital of West China hospital, Sichuan University, Mianzhu, Sichuan Province, China.

**Keywords:** HBV DNA, hepatocellular carcinoma, microvascular invasion, prognosis

## Abstract

To discuss the prognostic correlation between hepatitis B virus DNA (HBV DNA) level and HBV-related hepatocellular carcinoma (HCC) patients with microvascular invasion (MVI).

Data from HCC patients undergoing hepatectomy with pathological evidence of MVI were retrospectively collected and 1:1 propensity scoring matching (PSM) analysis was performed. According to the HBV DNA levels before and after surgery, the disease-free survival (DFS) and overall survival (OS) were evaluated using the Kaplan–Meier method, and the Cox proportional hazards regression was used to analyze the risk factors associated with the postoperative prognosis. After 1:1 PSM, 139 pairs of patients were enrolled in the high preoperative HBV DNA level group (H group) and low preoperative HBV DNA level group (L group), and after operation, patients with high preoperative HBV DNA levels were divided into the persistently high HBV DNA level group (P group) and the decreased HBV DNA level group (D group).

According to the multivariate analysis, the HBV DNA level of 2000 IU/ml or greater before operation was significantly associated with the DFS (hazard ratio, 1.322; 95%CI, 1.016–1.721) and OS (hazard ratio, 1.390; 95%CI, 1.023–1.888). A persistent HBV DNA level of 2,000 IU/ml or greater after operation was also the independent risk factor of DFS (hazard ratio, 1.421; 95%CI, 1.018–1.984) and OS (hazard ratio, 1.545; 95%CI, 1.076–2.219).

For the HBV-related HCC patients with MVI, preoperative high HBV DNA copies are prognostication of poorer prognosis, and effective antivirus treatment would significantly improve the patients’ prognosis.

## Abstract 2

Supplemental Digital Content is available in the text

## 1 Introduction

In 2018, liver cancer was reported to be the fourth leading cause of cancer death worldwide, with approximately 841,000 new cases and 782,000 deaths annually.^[1]^ As the most common primary liver cancer, hepatocellular carcinoma (HCC) is an important medical problem worldwide.^[2]^ Selective surgical intervention is one of the safest radical therapy methods at present.^[1,3]^ Unfortunately, the high rate of recurrence is still an issue plaguing potential curative treatment for HCC.^[4]^ As a marker of aggressive biological tumor behavior, the presence of microvascular invasion is regarded as a significant risk factor of the disease prognosis, especially for patients receiving potential curative therapy.^[5]^ Similarly, the recurrence of HCC is thought to be closely related to hepatitis B virus (HBV),^[6]^ and the level of HBV viral load is reported associated with the recurrence of HBV-related HCC.^[7,8]^

A considerable amount of research has reported that microvascular invasion (MVI) and hepatitis B virus (HBV) infection were risk factors for early and late recurrence after surgery, respectively.^[9–12]^ Studies show the relationship between the 2 risk factors. By enhancing the expression of transfer-related protein 1, HBV infection may enhance the angiogenesis process.^[13,14]^ HBV infection may weaken immune response to tumor cells, which provides an appropriate environment for the formation of MVI. In addition, some clinical research verified that infection and replication of HBV can promote the formation of MVI.^[15–17]^ Based on these facts, HBV infection and MVI are closely related to each other.

Several studies have reported on the prediction of MVI.^[5,18]^ However, for patients who have been pathologically diagnosed with MVI after surgery, Cheng et al and Jia et al reported postoperative adjuvant preventive TACE could improve the prognosis,^[19,20]^ while little attention has been paid on antivirus treatment. Because of the close relationship between HBV and MVI, we attempted to explore the prognosis of HCC patients with MVI from the perspective of HBV. These subjects should be extensively explored to determine whether the preoperative level of HBV viral load is related to the prognosis and whether effective antiviral therapy before and after operation is an adjuvant therapy to improve the prognosis of MVI. This study mainly aimed to resolve these problems by comparing patients’ DFS and OS to draw conclusions.

## 2 Methods

### 2.1 Study subjects

A retrospective analysis of 469 HCC patients who underwent curative liver resection in a West China hospital from January 2008 to December 2016 was conducted. The diagnostic criteria for preoperative liver cancer are based on the criteria of the American Association for the Study of Liver Diseases.^[21]^ All patients were pathologically confirmed with MVI, and the definition of MVI is: microscopic tumor invasion identified in portal or hepatic veins of the surrounding liver tissue, which was contiguous to the tumor.^[22]^ All patients underwent testing of HBV DNA levels preoperatively and postoperatively. The patients were divided into 2 groups according to the preoperative HBV DNA levels. To avoid selection bias, we performed 1:1 propensity score matching (PSM) analysis between the 2 cohorts. After PSM, 139 patients were included in each group. The criteria for inclusion and exclusion were as follows:

Inclusion criteria:

1. no previous HCC-related treatment,
2. without other malignant tumor history,
3. curative resection was performed,
4. functional liver status of Child-Pugh A or B,
5. pathologic confirmation of MVI patients;

Exclusion criteria:

1. patients who relapse or die within 30 days after surgery,
2. patients infected with hepatitis C virus,
3. patients with macrovascular invasion.

Our study has been approved by the West China Hospital of Sichuan University Biomedical Research Ethics Committee.

### 2.2 Treatment intervention

We evaluated the patients’ condition before undergoing operation. When preoperative examinations showed that the tumor was resectable and the liver function is sufficient to meet the needs of postoperative patients, hepatectomy was performed. Tumors were assessed by intraoperative ultrasound, including tumor size, number, location, and relation of the tumor to vascular structures. Liver resection was performed by a clamp-crushing method. The definition of curative resection is complete excision of the tumor with clear microscopic margin and no residual tumors demonstrated by computed tomography (CT) scan or angiography at 1 month after surgery.^[23]^ The serum HBV DNA levels were detected preoperatively and postoperatively using a real-time quantitative polymerase chain reaction (PCR) method. Reagent used for HBV DNA quantification was Quantitative Detection Kit for Hepatitis B Virus Nucleic Acid (PCR-Fluorescent Probe Method). The linear range was 20 IU/ml−2.0 × 10^9^ IU/ml and the detection instrument was SLAN-96P. Samples with results less than 100 IU/ml would be suggested re-measure by COBAS TaqMan HBV Test reagent, which was a more accurate reagent recommend by international Chronic Hepatitis B guidelines,^[24,25]^ and the linear range was 2.00E+01 IU/ml to 1.70E+08 IU/ml. Patients with high preoperative viral load received anti-virus treatment, and antiviral drugs such as entecavir (0.5 mg/day) and tenofovir were administered. If the viral load was not effectively controlled by entecavir, treatment protocols were changed to tenofovir.

### 2.3 Design

Before undergoing surgery, the HBV DNA levels of patients were measured and according to the HBV DNA level (≥2000 IU/ml or not), patients were divided into the H group (HBV DNA level ≥2000 IU/ml) and the L group (HBV DNA level <2000 IU/ml). To overcome selection bias, 1:1 PSM analysis was performed. Patients in the H group received antiviral therapy. HBV DNA levels were detected again after surgery. Based on the postoperative HBV DNA levels (≥2000 IU/ml or not), patients with high HBV DNA levels before operation were divided into the D group (HBV DNA level <2000 IU/ml) and P group (HBV DNA level ≥2000 IU/ml). Disease-free survival (DFS) and overall survival (OS) were compared among these groups with the Kaplan–Meier method, and significant differences were identified using log-rank analysis. Univariate and multivariate Cox proportional hazards regression analysis were performed to investigate the risk factors of poor prognosis.

### 2.4 Follow-up and survival analysis

Serum HBV DNA level, abdominal ultrasound, and alpha fetoprotein (AFP) were regularly reviewed every 3 months after operation. If suspicious recurrence lesions were found, contrast-enhanced computed tomography and enhanced magnetic resonance imaging were performed for further evaluation. The end points of follow-up were OS and DFS. OS is defined as the time from the date of surgery to the patient's death or the last follow-up. DFS is defined as the time from the date of surgery to the time of tumor recurrence.

### 2.5 Statistical analysis

Categorical variables were compared using the Chi-Squared (χ^2^) test or Fisher exact test. Continuous variables were compared using the unpaired *t* test or Mann–Whitney *U* test. Survival was analyzed using the Kaplan–Meier method, and survival curves were compared using the log-rank test. Univariate analyses were carried out using a Cox proportional hazards stepwise model to identify independent factors related to OS and DFS. The significant variables (*P* < .05) were subjected in the stepwise multivariate analysis. To overcome possible selection bias, 1:1 PSM between the H group and L group was applied using the nearest neighbor-matching method based on the clinical variables including age, sex, presence of diabetes, serum test (alanine aminotransferase (ALT) level, aspartate aminotransferase (AST) level, total bilirubin (TBIL) level, lymphocyte (LYM) count, and white blood cell (WBC) count), preoperative level of tumor marker (alpha fetoprotein (AFP)), tumor characteristics (number, diameter, encapsulation, differentiation, relationship with adjacent organs and liver capsule, presence of lymphatic metastasis, satellite nodules), and resection methods (anatomic resection or not).^[26]^ All analyses were performed using SPSS Statistics version 22.0 for Windows (IBM Corp), and all figures were created by GraphPad Prism 7.04 for Windows.

## 3 Results

### 3.1 Patient Characteristics

A total of 469 HCC patients who received hepatectomy with MVI from January 2008 to December 2016 were retrospectively analyzed. Among these, 319 patients meeting the criteria were selected for comparison. Patients were excluded from the final analysis if they had missing data (n = 121), had other malignancies (n = 1), recurred within 4 weeks (n = 3), were pathologically confirmed with mixed-type HCC (n = 1), or were lost to follow-up evaluation (n = 25). Finally, 319 patients (166 high preoperative HBV DNA level patients and 153 low preoperative HBV DNA level patients) were enrolled in the analysis. As shown in Supplementary Table 1a, the baseline characteristic data before PSM analysis showed significant differences, including ALT level (*P* = .038), AST level (*P* = .041), invasion of liver capsule (*P* = .011) and tumor well differentiation (*P* = .007), respectively. After 1:1 PSM with a caliper of 0.1, as shown in Supplementary Table 1b, there were 139 patients in each group with comparable baseline characteristics. Patients in the P and D groups had comparable basic characteristics (Supplementary Table 1c).

### 3.2 Association of Preoperative HBV DNA Level with Prognosis

**Figure 1:**
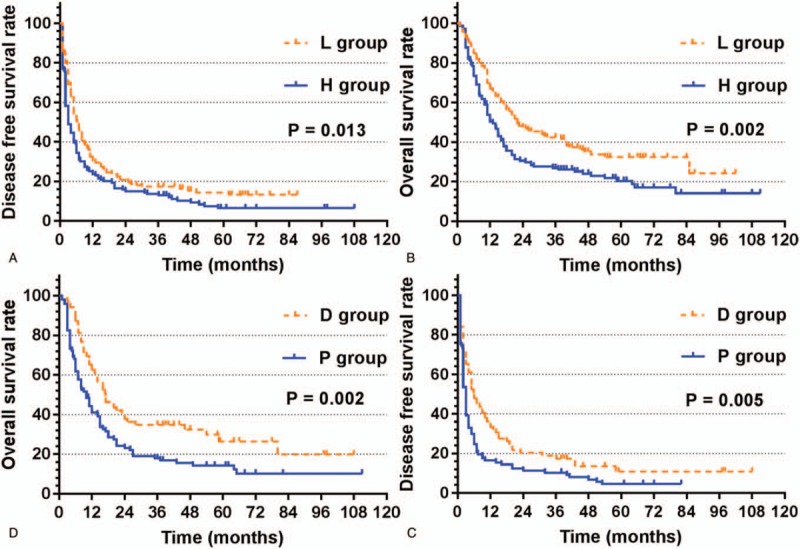
Kaplan–Meier analysis of disease-free survival (DFS) and overall survival (OS) for hepatocellular carcinoma (HCC) patients with microvascular invasion (MVI). (A) DFS for high and low HBV DNA levels before operation; (B) OS for high and low HBV DNA levels before operation; (C) DFS for high and low HBV DNA levels after operation; (D) OS for high and low HBV DNA levels after operation.

During the follow-up, 110 patients in the H group died, while 91 patients in the L group died, and there were 128 recurrences in the H group and 119 recurrences in the L group. For patients in the H group, 1-, 2-, 3-, and 5-year recurrence rates after surgery were 76.3, 84.9, 86.3, and 93.5%, while for L group patients, the recurrence rates were 69.8, 79.1, 82.7, and 85.7%, respectively (*P* = .013) (Fig. 1A). Patients in the H group had significantly worse overall survival rate than patients in the L group. The 1-, 2-, 3-, and 5-year survival rates were 50.3, 30.6, 26.9, and 20.4% vs 66.9, 47.5, 42.4, and 32.4%, respectively (*P* = .002) (Fig. 1B). We performed multifactorial analysis of both groups (Table 1), and found that lymphocyte count decrease the risk of recurrence (hazard ratio, 0.787; 95% confidence interval (CI), 0.629–0.986), and in addition to incomplete tumor encapsulation (hazard ratio, 1.668; 95% CI, 1.266–2.198) and invasion of the liver capsule (hazard ratio, 1.355; 95% CI, 1.027–1.788), a HBV DNA level of 2000 IU/ml or greater was the risk factor of DFS (hazard ratio, 1.354; 95%CI, 1.050–1.745). As for OS, multifactorial analysis indicated lymphocyte count decrease the risk of death (hazard ratio, 0.764; 95%CI, 0.596–0.981), and except HBV DNA level of 2000 IU/ml or greater (hazard ratio, 1.499; 95% CI, 1.130–1.987), incomplete tumor encapsulation (hazard ratio, 1.808; 95% CI, 1.327–2.464), poor differentiation (hazard ratio, 1.379; 95% CI, 1.038–1.832) and high serum AST level (hazard ratio, 1.004; 95% CI, 1.002–1.007) were also risk factors of OS.

**Table 1:**
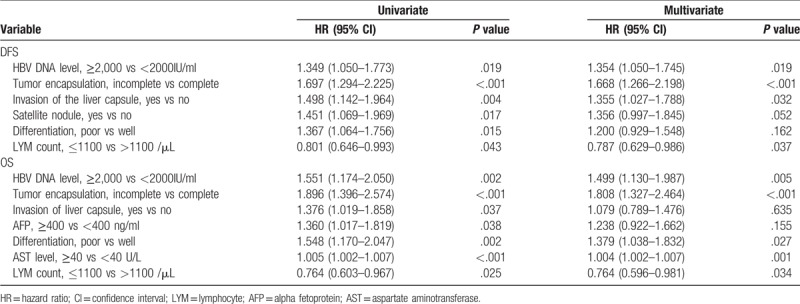
Uni- and multivariate analyses of disease-free survival (DFS) and overall survival (OS) for patients before operation.

### 3.3 Association of postperative HBV DNA levels in the H group with prognosis

The patients with preoperative high HBV DNA levels received antiviral therapy before operation, and then, tow various situations involving possibilities were investigated: based on the level of HBV DNA after operation (≥2000 IU/ml or less), this cohort of patients was divided into 2 groups: D group and P group. For P group patients, 1-, 2-, 3-, and 5-year recurrence rates after surgery were 83.5, 88.7, 89.7, and 95.4%, while D group, the recurrence rates were 66.7, 79.7, 81.2, and 89.2%, respectively (*P* = .005) (Fig. 1C). This trend was also found in OS; for P group patients, the 1-, 2-, 3-, and 5-year OS rates after surgery were 41.4, 23.2, 17.9, and 14.3%, while D group, the OS were 62.3, 37.7, 34.8, and 26.5% (*P* = .002) (Fig. 1D). We found a persistent HBV DNA level of 2000 IU/ml or greater was the independent risk factor of DFS (hazard ratio, 1.421; 95% CI, 1.018–1.984) and OS (hazard ratio, 1.545; 95% CI, 1.076–2.219) (Table 2).

**Table 2:**
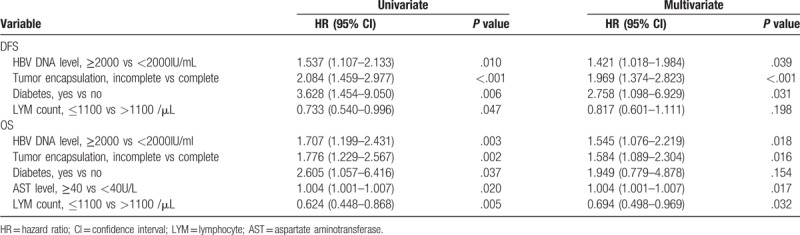
Uni- and multivariate analyses of disease-free survival (DFS) and overall survival (OS) for patients with preoperatively high HBV DNA level after operation.

## 4 Discussion

Our research indicated that patients with high preoperative HBV DNA levels have poorer prognosis compared with those with low preoperative HBV DNA levels. In addition, for the patients with high preoperative HBV DNA levels, persistent high HBV DNA levels after surgery also leads to poor prognosis results, suggesting that high HBV DNA loading plays a dismal role for prognosis. These results are in accordance with the reports of other studies.^[6,27]^

Unlike many other studies, the scope of this research lies in patients with background of MVI. In HCC, vascular invasion can be either macroscopic with microscopic vascular invasion or microscopic alone.^[28]^ Microscopic venous invasion is frequent and independently related to post-resectional outcome.^[29]^ There has been an increasing interest in the relationship between MVI and hepatitis B virus. With the analysis of clinical data from 45 HCC specimens, Moon et al reported that high expression level of metastasis-associated protein 1 (MTA1) was associated with vascular invasion.^[30]^ In addition, Xu et al found that MTA1 plays a critical role in invasion and metastasis of tumor in HBV-related HCC,^[31]^ and this result is consistent with another study indicating that a positive cross-talk exists between HBx and MTA1, which is an important factor in angiogenesis and metastasis.^[32]^ In patients with HBV–HCC, the expression of MTA1 in HCCs is reported closely related to microvascular invasion.^[14]^ Many studies have demonstrated that active HBV replication was associated with the development of vascular invasion.^[15,31]^

In the highly endemic Asia–Pacific region, more than half of HCC cases are associated with hepatitis B virus infection.^[33]^ Li et al found that preoperative antiviral treatment can reduce the formation of MVI and the recurrence after hepatectomy.^[34]^ Sun et al reported that for patients with MVI, postoperative transcatheter arterial chemoembolization has a positive effect on prognosis.^[19]^ Our research confirmed that with the backgrounds of MVI, the prognosis of patients with high preoperative HBV DNA levels was worse than those of low ones, and effective antivirus treatment contributes to better prognosis. Antiviral therapy is another effective treatment for preventing recurrence in patients with MVI.

Our research has several limitations. First, it should be noted that only patients diagnosed with MVI were enrolled in our research, while those without MVI require further discussion. Second, our data was retrospectively collected from a single medical center. Third, for patients with low HBV DNA levels before operation, we did not analyze the postoperative condition because the elevation of HBV DNA level sometimes occurred after operation.

## 5 Conclusion

For the HBV-related HCC patients with MVI, an HBV DNA level of 2000 IU/ml or greater before operation indicates a poorer prognosis, and effective antivirus treatment would significantly improve the patients’ prognosis.

## 6 Data availability

The datasets generated and analyzed during the current study are available from the corresponding author upon reasonable request.

## Acknowledgments

This work was supported by grants from the National Natural Science Foundation of China (No. 71673193) and the Key Technology Research and Development Program of the Sichuan Province (2015SZ0131 and 2017FZ0082).

## Author contributions

M.Z and B.L conceived and designed the study; L.L was responsible for the analysis and interpretation of data and wrote the paper.

**Conceptualization:** Bo Li, Ming Zhang.

**Data curation:** Lian Li.

**Formal analysis:** Lian Li.

**Funding acquisition:** Ming Zhang.

**Methodology:** Bo Li, Ming Zhang.

**Project administration:** Ming Zhang.

**Software:** Lian Li.

**Supervision:** Bo Li, Ming Zhang.

**Validation:** Bo Li.

**Writing – original draft:** Lian Li.

## Supplementary Material

Supplemental Digital Content
