# Supplementary material for: HBV DNA levels impact the prognosis of hepatocellular carcinoma patients with microvascular invasion
Source: Medicine (Baltimore). 2019 Jul 5;98(27):e16308. doi: 10.1097/MD.0000000000016308 (PMC6635265; doi:10.1097/MD.0000000000016308)
Supplement: Supplemental Digital Content [file medi-98-e16308-s001.doc]

| **Supplementary Table 1a** |  | | | |
| --- | --- | --- | --- | --- |
| **Baseline characteristics before propensity score matching** | | | | |
| **Variable** | | **H group** | **L group** | **P value** |
|  |  | **n=166** | **n=153** |  |
| Age(<50y) | | 91(54.8%) | 72(47.1%) | 0.166 |
| Gender(male) | | 147(88.6%) | 132(86.3%) | 0.712 |
| Tumor diameter(<5cm) | | 32(19.3%) | 43(28.1%) | 0.063 |
| Tumor number(single) | | 120(72.3%) | 117(76.5%) | 0.393 |
| Incomplete tumor encapsulation | | 108(65.1%) | 101(66.0%) | 0.858 |
| Diabetes | | 5(3.0%) | 10(6.5%) | 0.137 |
| AFP( <400ng/mL) | | 56(33.7%) | 65(42.5%) | 0.108 |
| Invading adjacent organs | | 17(10.2%) | 15(9.8%) | 0.897 |
| Anatomic resection | | 69(41.6%) | 54(35.3%) | 0.250 |
| Well differentiation | | 65(39.2%) | 83(54.2%) | 0.007 |
| Invasion of liver capsule | | 42(25.3%) | 59(38.6%) | 0.011 |
| Satellite nodules | | 34(20.5%) | 30(19.6%) | 0.846 |
| Lymphatic metastasis | | 5(3.0%) | 4(2.6%) | 0.830 |
| Cirrhosis | | 92(55.4%) | 72(47.1%) | 0.135 |
| ALT level (U/L)(IQR) | | 42.0(31.0-71.3) | 38.0(27.0-59.0) | 0.038 |
| AST level (U/L)(IQR) | | 55.0(34.7-82.3) | 45.0(33.5-64.0) | 0.041 |
| TBIL level (mmol/L)(IQR) | | 14.5(11.3-19.1) | 14.3(10.5-19.1) | 0.632 |
| LYM count (10 9 /L)(IQR) | | 1.3(1.0-1.7) | 1.4(1.0-1.9) | 0.212 |
| WBC count (10 9 /L) (IQR) | | 5.5(4.7-6.7) | 5.3(4.4-7.1) | 0.555 |

Abbreviations: H group, high preoperative HBV DNA level group; L group, low preoperative HBV DNA level group; AFP, alpha fetoprotein; ALT, alanine aminotransferase; IQR, interquartile range; AST, aspartate aminotransferase; TBIL, total bilirubin; LYM, lymphocyte; WBC, white blood cell

| **Supplementary Table 1b** |  | | | |
| --- | --- | --- | --- | --- |
| **Baseline characteristics after propensity score matching** | | | | |
| **Variable** | | **H group** | **L group** | **P value** |
|  |  | **n=139** | **n=139** |  |
| Age(<50y) | | 77(55.4%) | 66(47.5%) | 0.187 |
| Gender(male) | | 125(89.9%) | 119(85.6%) | 0.532 |
| Tumor diameter(<5cm) | | 31(22.3%) | 36(25.9%) | 0.483 |
| Tumor number(single) | | 99(71.2%) | 104(74.8%) | 0.499 |
| Incomplete tumor encapsulation | | 90(64.7%) | 91(65.5%) | 0.900 |
| Diabetes | | 5(3.6%) | 5(3.6%) | 1.000 |
| AFP( <400ng/mL) | | 48(34.5%) | 55(39.6%) | 0.385 |
| Invading adjacent organs | | 11(7.9%) | 13(9.4%) | 0.669 |
| Anatomic resection | | 57(41.0%) | 52(37.4%) | 0.539 |
| Well differentiation | | 63(45.3%) | 74(53.2%) | 0.187 |
| Invasion of liver capsule | | 39(28.1%) | 53(38.1%) | 0.074 |
| Satellite nodules | | 30(21.6%) | 26(18.7%) | 0.550 |
| Lymphatic metastasis | | 4(2.9%) | 4(2.9%) | 1.000 |
| Cirrhosis | | 74(53.2%) | 68(48.9%) | 0.472 |
| ALT level (U/L)(IQR) | | 40.0(31.0-66.0) | 38.0(27.0-59.0) | 0.138 |
| AST level (U/L)(IQR) | | 49.0(33.0-78.0) | 46.0(34.0-64.0) | 0344 |
| TBIL level (mmol/L)(IQR) | | 14.4(11.3-18.2) | 14.1(10.4-18.6) | 0.740 |
| LYM count (10 ^9^ /L) | | 1.4±0.6 | 1.5±0.7 | 0.604 |
| WBC count (10 ^9^ /L) | | 5.7±1.6 | 5.7±2.4 | 0.929 |

Abbreviations: H group, high preoperative HBV DNA level group; L group, low preoperative HBV DNA level group; AFP, alpha fetoprotein; ALT, alanine aminotransferase; IQR, interquartile range; AST, aspartate aminotransferase; TBIL, total bilirubin; LYM, lymphocyte; WBC, white blood cell

| **Supplementary Table 1c** |  | | | |
| --- | --- | --- | --- | --- |
| **Baseline characteristics of patients with preoperatively high HBV DNA level after operation** | | | | |
| **Variable** | | **P group** | **D group** | **P value** |
|  |  | **n=97** | **n=69** |  |
| Age(<50y) | | 52(53.6%) | 39(56.5%) | 0.710 |
| Gender(male) | | 84(86.6%) | 63(91.3%) | 0.348 |
| Tumor diameter(<5cm) | | 16(16.5%) | 16(23.2%) | 0.281 |
| Tumor number(single) | | 72(74.2%) | 48(69.6%) | 0.508 |
| Incomplete tumor encapsulation | | 65(67.0%) | 43(62.3%) | 0.532 |
| Diabetes | | 5(5.2%) | 0(0.0%) | 0.055 |
| AFP( <400ng/mL) | | 29(29.9%) | 27(39.1%) | 0.215 |
| Invading adjacent organs | | 9(9.3%) | 8(11.6%) | 0.628 |
| Anatomic resection | | 44(45.4%) | 25(36.2%) | 0.240 |
| Well differentiation | | 40(41.2%) | 25(36.2%) | 0.515 |
| Invasion of liver capsule | | 25(25.8%) | 17(24.6%) | 0.868 |
| Satellite nodules | | 18(18.6%) | 16(23.2%) | 0.466 |
| Lymphatic metastasis | | 1(1.0%) | 4(5.8%) | 0.077 |
| Cirrhosis | | 55(56.7%) | 37(53.6%) | 0.694 |
| ALT level (U/L)(IQR) | | 42.0(31.0-62.5) | 41.0(31.0-79.0) | 0.842 |
| AST level (U/L)(IQR) | | 55.0(36.0-82.5) | 55.0(32.5-83.5) | 0.826 |
| TBIL level (mmol/L)(IQR) | | 13.8(11.3-19.0) | 15.1(11.6-19.7) | 0.451 |
| LYM count (10 ^9^ /L)(IQR) | | 1.3(1.0-1.6) | 1.3(1.1-1.8) | 0.262 |
| WBC count (10 ^9^ /L)(IQR) | | 5.6(4.7-6.8) | 5.3(4.5-6.7) | 0.439 |

Abbreviations: P group, persistently high HBV DNA level group; D group, decreased HBV DNA level group; AFP, alpha fetoprotein; ALT, alanine aminotransferase; IQR, interquartile range; AST, aspartate aminotransferase; TBIL, total bilirubin; LYM, lymphocyte; WBC, white blood cell
